# Adherence to Guideline‐Recommended cancer screening among Utah cancer survivors

**DOI:** 10.1002/cam4.5168

**Published:** 2022-08-27

**Authors:** Morgan M. Millar, Sandra L. Edwards, Kimberly A. Herget, Brian Orleans, Blessing S. Ofori‐Atta, Anne C. Kirchhoff, Marjorie E. Carter, Marie Nagata, Carol Sweeney

**Affiliations:** ^1^ Utah Cancer Registry University of Utah Salt Lake City Utah USA; ^2^ Division of Epidemiology University of Utah Salt Lake City Utah USA; ^3^ Huntsman Cancer Institute University of Utah Salt Lake City Utah USA; ^4^ Department of Pediatrics University of Utah Salt Lake City Utah USA; ^5^ Breast and Cervical Cancer Early Detection Program, Utah Department of Health and Human Services Salt Lake City Utah USA

**Keywords:** breast neoplasms, cancer survivors, cervical neoplasms, colorectal neoplasms, early detection of cancer, registries, surveys and questionnaires

## Abstract

**Background:**

Adherence to cancer screening is important for cancer survivors because they are at high risk of subsequent cancer diagnoses or recurrence. We assessed adherence to breast, cervical, and colorectal cancer‐(CRC)‐screening guidelines and evaluated demographic disparities among a population‐based sample of survivors.

**Methods:**

A representative sample of Utah survivors diagnosed from 2012–2018 with any reportable invasive cancer was selected from central cancer registry records for a survey about survivorship needs. We estimated the proportion of eligible survivors adhering to U.S. Preventive Services Task Force screening guidelines and calculated risk ratios and 95% confidence intervals. Analyses were age‐adjusted and weighted to account for sample design and nonresponse.

**Results:**

And 1421 survivors completed the survey (57.2% response rate). Screening adherence was 74.4% for breast, 69.4% for cervical, and 79.7% for CRC. Rural residents were more likely to adhere to breast cancer screening than urban residents (86.1% vs. 72.7%; adjusted RR = 1.19, CI = 1.05, 1.36). Higher educational attainment was associated with increased adherence to cervical and colorectal cancer screening. Younger age was associated with greater adherence to cervical cancer screening (*p* = 0.006) but lower adherence to CRC screening (*p* = 0.003). CRC screening adherence was lower among the uninsured and those without a primary care provider (45.6%) compared to those with a regular provider (83.0%; adjusted RR = 0.57, CI = 0.42, 0.79).

**Conclusions:**

Surveys based on samples from central cancer registries can provide population estimates to inform cancer control. Findings demonstrate work is needed to ensure all Utah cancer survivors obtain recommended cancer screenings. Efforts should focus particularly on increasing uptake of breast and cervical cancer screening and reducing demographic disparities in CRC screening.

**Precis:**

Despite high risk for subsequent cancer diagnosis, Utah cancer survivors are not all obtaining recommended breast, cervical, and colorectal cancer screenings. This presents a significant healthcare gap.

## Graphical abstract

In this paper, we examine adherence to breast, cervical, and colorectal cancer screening guidelines using a population‐based sample survey of cancer survivors in Utah. Our findings indicate additional work is needed to ensure all Utah cancer survivors obtain recommended cancer screenings and reduce demographic disparities in screening.

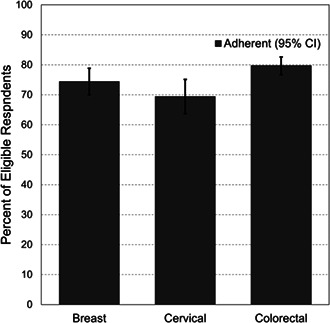

## 1 BACKGROUND

Cancer survivors are a growing population^1^, ^2^ in the United States. In 2019 there were nearly 17 million survivors living in the United States, with estimates that this population will grow to over 22 million by 2030.^3^ Cancer survivors not only are susceptible to recurrence of their original cancer but also have an increased risk of new primary cancers relative to the general population.^4^, ^5^, ^6^, ^7^ Yet not all survivors obtain recommended cancer screenings. There are also indications of disparities in cancer screening adherence among cancer survivors; for example, research has found those with higher education or higher income,^8^ and those of older age^9^, ^10^ are more likely than their counterparts to have undergone screening.

The goal of cancer screening is to reduce mortality by detecting cancer or its precursors at earlier, more treatable stages. The United States Department of Health and Human Services' Healthy People 2020 initiative, which set overall health goals with measurable objectives, aimed to increase utilization of cancer screening in the United States among all age‐eligible adults.^11^ Healthy People 2020 is an initiative that provides science‐based, 10‐year national objectives for improving health among the United States population. Its mission is to identify nationwide health improvement priorities, increase awareness, provide measurable objectives and goals, engage multiple stakeholders to take action to strengthen policies, and identify critical research and evaluation needs. The specific targets set by Healthy People 2020 for screening guideline adherence in the targeted age groups were 93.0% for cervical cancer, 70.5% for colorectal cancer, and 81.1% for breast cancer. However, cancer screening remains underutilized in the United States and by 2020, fell short of the Healthy People targets.^12^, ^13^, ^14^ Goals for the new iteration, Healthy People 2030, also include increasing the proportion of adults who receive cancer screenings in accordance with guidelines. Disparities in keeping up to date with cancer screening persist, including by income, educational attainment, race, ethnicity, age, and other factors,^14^ and increasing screening uptake remains a priority. Yet not much attention has been given to the screening behaviors of the cancer survivor population.

Cancer control programs, including the Centers for Disease Control and Prevention's National Comprehensive Cancer Control Program, have recognized the importance of assessing the unique healthcare needs and experiences of cancer survivors. This program provides funding, guidance, and assistance to state and territorial cancer control programs across the country to develop, implement, and evaluate statewide cancer control plans.^15^ The Utah Comprehensive Cancer Control Program, in coordination with a diverse group of stakeholders through the Utah Cancer Action Network, developed the 2016–2020 Utah Comprehensive Cancer Prevention and Control Plan as a guide for those involved in the planning, implementation, and evaluation of cancer control efforts.^16^, ^17^ This plan included a priority area of focus for survivorship quality of life. One topic of interest is ensuring cancer survivors obtain all recommended preventive care, including cancer screenings.

While major surveys such as the Behavioral Risk Factors Surveillance System^18^ are used in tracking cancer‐screening utilization in the general public both nationally and at the state level, such surveys have limitations for assessing the health status of recent cancer survivors. Central cancer registries provide a high‐quality sample frame for obtaining population‐based samples of cancer survivors in order to describe survivors' experiences and assess health needs. We utilize data from a multi‐year survey of recent cancer survivors in Utah to evaluate cancer screening adherence. The purpose of this study was to assess what proportion of cancer survivors are obtaining recommended routine screenings for breast, cervical, and colorectal cancer. Additionally, we sought to identify whether demographics (age, ethnicity, education, rural location), healthcare‐related characteristics such as insurance access or having a primary care provider, or cancer characteristics including site and stage at diagnosis are associated with adherence to screening guidelines.

## 2 METHODS

### 2.1 Study population and sample design

We assessed screening adherence among cancer survivors using responses to a survey conducted to understand survivorship experiences and health needs among individuals diagnosed with cancer from 2012 through 2018 in Utah. Eligible living survivors were identified using records from the Utah Cancer Registry, a population‐based registry that collects and maintains information on all reportable cancer diagnoses in Utah. Utah Cancer Registry data are complete and of high quality according to the standards of the U.S. National Cancer Institute Surveillance, Epidemiology, and End Results program and the U.S. Centers for Disease Control and Prevention National Program of Cancer Registries. Eligibility criteria for the survey included age 18 or older at time of diagnosis and Utah residency at time of diagnosis and at the time of the survey. Survivors of any reportable, invasive cancer diagnosis were eligible to be sampled for the study. No cancer sites were excluded. The 2018 sample also included benign brain or central nervous system tumor diagnoses, but in subsequent years benign diagnoses were excluded. This study was reviewed by the Utah Department of Health Institutional Review Board. Participants were informed that completing the survey signified consent to participate.

To support inference of the survey results to populations with potential health disparities, a probability‐based sample was stratified based on an area‐level measure of health insurance coverage and on Hispanic ethnicity. Survivors who were of Hispanic ethnicity or were residents of Small Health Statistical Areas (geographic areas defined by the Utah Department of Health)^19^ with low health insurance coverage were assigned a higher sampling probability. Low insurance coverage was defined as areas below the median proportion of insured residents, based on data obtained from responses to the Utah Behavioral Risk Factors Surveillance System survey, described below.^20^ A table identifying the sample frame and sample probabilities is available as a Table S1.

### 2.2 Survey

The questionnaire was designed to support evaluation of cancer survivorship issues targeted in the Utah Comprehensive Cancer Prevention and Control Plan^16^ and included measures of general health status, health behaviors, healthcare, cancer treatment, as well as questions about financial impacts of cancer treatment, caregivers, and social connectedness. When possible, we utilized existing questions from the Behavioral Risk Factors Surveillance System survey or other validated instruments. The Behavioral Risk Factors Surveillance System is one of the primary health‐related surveys in the United States, providing data on health‐related risk behaviors, chronic health conditions, and utilization of preventive health services at local, state, and nationwide levels.^18^ As it is a primary source of data for assessing cancer‐screening adherence in the general population, we used questions derived from this survey in our study. In 2019–2020, our study questionnaire was available in both English and Spanish. The questionnaire was created in both paper and web formats, with the web instrument using Qualtrics software. The paper version of the study questionnaire in its entirety, including the wording of all survey questions and response options, is included as an Appendix S1 for this manuscript.

The survey was conducted in 2018, 2019, and 2020 using a mixed‐mode, push‐to‐web methodology^21^ for survivors under age 80, and a paper‐only response method for survivors aged 80 or above. The push‐to‐web method entails contacting individuals by postal mail to request response to an online questionnaire when email addresses are not available. The contact sequence was designed according to recommendations in the literature^22^ and used postal mailings and phone calls. It began with a pre‐notification letter with a brochure about the registry, followed by a formal invitation letter with either the survey web address or a paper questionnaire and stamped return envelope for those age 80 or above. The initial invitation also included a $2.00 cash pre‐incentive. A reminder letter, a packet containing a replacement questionnaire or a first paper questionnaire and stamped return envelope, and then a phone call follow‐up were used to reach those who did not respond to initial contacts.

### 2.3 Measures

Cancer diagnosis information and certain demographic variables were obtained from cancer registry records. These included cancer site, stage at diagnosis, year of diagnosis, current age at time of survey, and place of residence at the time of diagnosis. Race and ethnicity information were based on participants' self‐reported responses when available, and registry records otherwise. Information about current insurance, current primary care provider, educational attainment, financial hardship related to cancer diagnosis, and current general health was gathered via the questionnaire. To measure adherence to cancer screening, the survey included a series of questions asking long it has been since the respondent has had a mammogram, pap test, colonoscopy, sigmoidoscopy, and stool test, if ever. Participants who reported having a pap test were also asked whether their last test included HPV testing. We worded these items to match the questions as asked in the Behavioral Risk Factors Surveillance System Survey questionnaire.

Using responses to the cancer‐screening questions, we created variables representing having received screening according to U.S. Preventive Services Task Force recommendations for cancer screening for breast,^23^ cervical,^24^ and colorectal cancer.^25^ For each screening type, analysis of adherent versus non‐adherent was limited to those survivors who met the age and sex criteria for each respective guideline: women aged 50–74 for breast cancer screening, women aged 21–65 for cervical cancer screening, and men and women aged 50–75 for colorectal cancer screening. Our analysis of screening adherence included survivors of cancers of all sites.

Based on U.S. Preventive Services Task Force guidelines, for breast cancer screening, women were coded as adherent when reporting mammography within the past 2 years. For cervical cancer screening, a Pap test within the last 3 years represents adherence for women aged 21 to 29, and for women aged 30–65, adherence included pap screening within 3 years with cervical cytology alone, or between 3–5 years with cervical cytology and/or human papilloma virus (HPV) testing. For colorectal cancer, survivors were coded as adherent if they reported a stool test within the last year, a sigmoidoscopy in the last 5 years, or a colonoscopy within the last 10 years. Participants reporting a most recent screening test occurring outside of the timeframes defined as adherent, or reporting “not sure” or “never” for any screening were coded as non‐adherent.

To determine guideline adherence among survivors whose diagnosis was of the cancer site associated with each screening type, we consulted follow‐up care recommendations of the National Comprehensive Cancer Network.^26^ Women who had been diagnosed with breast cancer were coded as compliant if they reported a mammogram in the past year. Women who had been diagnosed with cervical cancer were considered compliant if they had received a pap test in the last year. We excluded colorectal cancer survivors (*n* = 57) from our assessment of adherence to colorectal cancer screening for two reasons. First, follow‐up screening recommendations for colorectal cancer are variable depending upon multiple clinical factors not measured in our survey. Second, because our sample consisted of survivors who had been diagnosed within the past ten years and adherence by colorectal cancer screening is assessed over a period as long as ten years, it is probable that colonoscopies reported by colorectal cancer survivors in the survey included those that diagnosed their cancer rather than during the survivorship period. This would not be the case with cervical or breast cancer survivors in our sample as all participants were surveyed more than one year after diagnosis.

### 2.4 Analysis

We estimated the proportion of survivors who reported having breast, cervical, and colorectal cancer screenings in accordance with U.S. Preventive Services Task Force guidelines for all eligible survivors. We also assessed whether adherence varied according to various demographic characteristics (age, Hispanic ethnicity, educational attainment, rural residence), healthcare‐related factors, and cancer variables including site and stage of diagnosis. Due to the demographics of the Utah cancer survivor population, we did not have enough survivors identifying as a race other than white to allow for analyses of adherence by race. We calculated crude and adjusted risk ratios and 95% confidence intervals to compare screening adherence across demographic subgroups. Adjusted models controlled for ethnicity, age, education, rural residence, and years from diagnosis. All analyses were weighted to account for the sample design and nonresponse, and age‐adjusted to the Utah adult cancer survivor population. Statistical analyses were conducted using SAS 9.4 and R.

## 3 RESULTS

**FIGURE 1:**
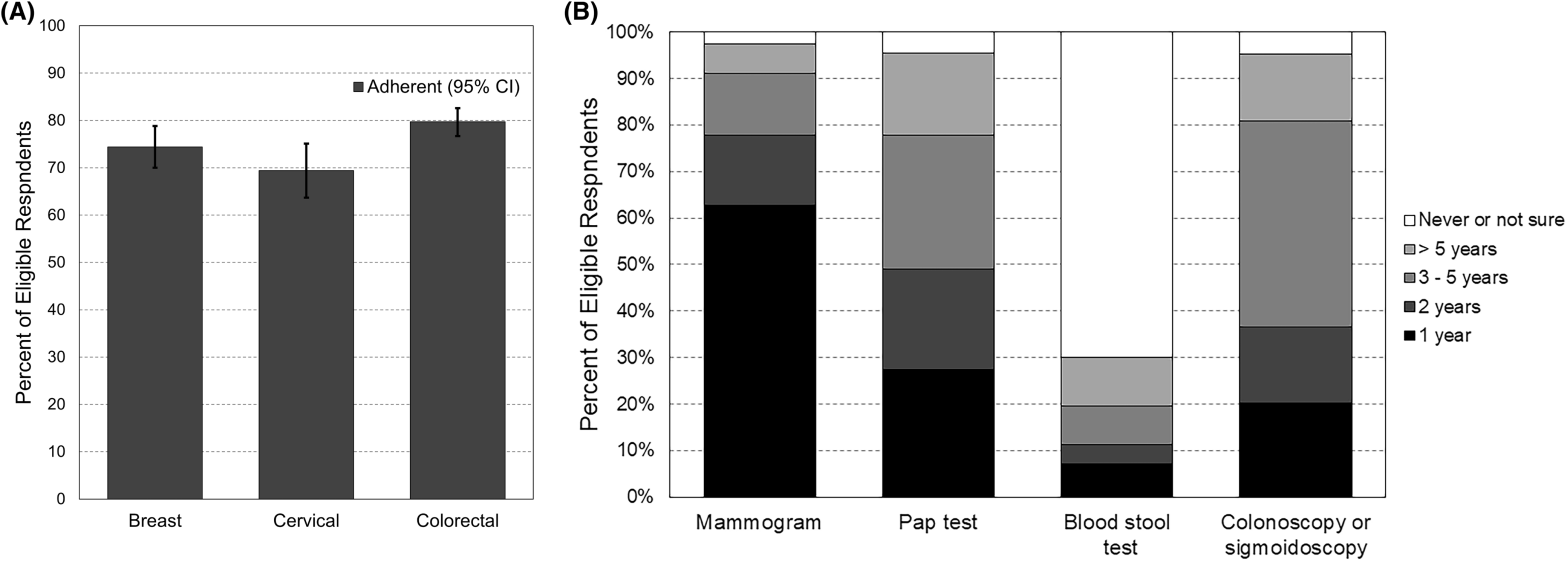
(A and B). Utah cancer survivors' (A) Adherence to cancer‐screening guidelines and (B) time since last cancer screening, 2018–2020. Percent of participants weighted to account for survey sample design and non‐response.

In total, 1421 of Utah survivors responded to the survey (57.2% response rate). The demographics of those who participated were similar to nonrespondents in regard to sex and rural residence, but older survivors and non‐Hispanic white survivors were over‐represented in the responding sample compared to the nonrespondents. Our analyses included 476 females age‐eligible for breast cancer screening, 311 females age‐eligible for cervical cancer screening, and 883 survivors (412 male, 471 female) for age‐eligible colorectal cancer screening (Table 1). Overall, 74.4% (95% confidence interval [CI] 70.0, 78.8) of eligible cancer survivors reported having received a mammography screening for breast cancer within the past two years (Figure 1). Adherence with cervical cancer screening among age‐eligible survivors was lower (69.4%, 95% CI 63.7, 75.1). Adherence with colorectal cancer screening was the most utilized of the three screening types, with adherence at 79.7% (95% CI 76.7, 82.6).

**TABLE 1:** Demographics of cancer survivors assessed for adherence to breast, cervical, and colorectal cancer screening guidelines; Utah cancer survivors surveyed 2018‐2020^a^

	Screening type					
	Breast		Cervical		Colorectal	
	n	%^b^	n	%^b^	n	%^b^
Female	476	100.0	311	100.0	471	52.4
Race and ethnicity						
Hispanic, any race	62	5.7	53	6.1	97	5.3
Non‐Hispanic white	404	91.1	243	86.6	770	91.9
Non‐Hispanic, other race	^	3.2	15	7.3	16	2.8
Age						
Under 45	n/a	—	59	24.4	n/a	—
45‐54^c^	65	13.8	73	24.8	92	10.9
55‐64^d^	220	44.7	179	50.9	330	36.2
65–75	191	41.4	—	—	461	52.9
Education						
High school or less	105	20.2	71	21.0	186	19.9
Some college	199	43.4	136	44.0	342	40.0
College graduate	167	36.5	101	34.9	338	40.2
Rural residence	66	12.8	51	15.3	130	12.9
Health insurance						
Uninsured/unknown	11	1.7	16	4.9	32	3.6
Insured	465	98.3	295	87.1	851	96.4
No primary care provider	38	6.9	41	12.7	59	6.3
Cancer site						
Breast	199	38.9	121	35.0	211	22.1
Cervical	^	1.5	^	2.9	^	0.9
Colorectal	28	5.9	18	5.9	—	—
Melanoma	54	11.6	39	13.8	128	14.6
Prostate	—	—	—	—	202	22.5
Other	189	42.1	126	42.4	335	39.8
Years since cancer diagnosis						
<2	61	12.6	39	11.9	101	10.7
2‐<4	193	41.1	123	39.4	376	42.4
4+	222	46.4	149	48.7	406	46.9

*Note:* ^Small cell count suppressed in accordance with confidentiality guidelines of the Utah Department of Health.

^a^ Participants of all cancer sites included.

^b^ Percent weighted to represent all eligible cancer survivors diagnosed in 2012–2018; weighting accounts for survey sample design and non‐response.

^c^ Breast and colorectal cancer‐screening samples are limited to ages 50 and above.

^d^ This category includes individuals aged 65 for cervical cancer sample only.

Adherence to breast cancer‐screening guidelines varied by geography and stage at diagnosis (Table 2). Survivors residing in rural areas had higher adherence (86.1%) compared to those in urban areas (72.7%; adjusted risk ratio [aRR]: 1.19, 95% CI: 1.05, 1.36). Individuals diagnosed at regional or distant stage were significantly less likely to adhere to screening guidelines than those diagnosed at local stage disease (regional stage: aRR: 0.79, 95% CI: 0.65, 0.96; distant stage: aRR: 0.76, 95% CI: 0.59, 0.98). No other demographic or cancer characteristics examined were significant predictors of adherence to breast cancer‐screening recommendations.

**TABLE 2:** Utah cancer survivors' adherence to breast cancer‐screening guidelines by demographic, cancer diagnosis, and healthcare characteristics

	Percent adherent	Crude risk ratio (RR)^b^			Adjusted risk ratio (RR)^c^		
	%^a^	RR	95% Confidence interval		RR	95% Confidence interval	
			Lower	Upper		Lower	Upper
Hispanic ethnicity							
Hispanic	78.6	1.06	0.90	1.25	1.05	0.89	1.24
Non‐Hispanic	74.1	Ref			Ref		
Age							
50–54	70.9	0.93	0.76	1.12	0.92	0.75	1.11
55–64	73.5	0.96	0.85	1.09	0.95	0.84	1.08
65–74	76.6	Ref			Ref		
Education							
High school or less	68.8	Ref			Ref		Ref
Some college or more	75.8	1.10	0.94	1.30	1.10	0.94	1.30
Geography							
Urban	72.7	Ref			Ref		
Rural	86.1	1.19	1.04	1.34	1.19	1.05	1.36
Area‐level proportion uninsured							
More uninsured	75.9	1.03	0.92	1.16	1.02	0.91	1.14
Fewer uninsured	73.5	Ref			Ref		
Health insurance							
Uninsured/unknown	52.8	0.71	0.38	1.31	0.79	0.43	1.46
Insured	74.8	Ref			Ref		
Financial hardships due to cancer							
Yes	71.0	0.93	0.82	1.05	0.94	0.82	1.07
No	76.4	Ref			Ref		
General health							
Good or better	76.2	Ref.					
Fair or poor	64.4	0.85	0.68	1.05	0.84	0.68	1.03
Has primary care provider							
Yes	74.7	Ref			Ref		
No	69.0	0.92	0.72	1.18	0.91	0.70	1.18
Cancer site							
Breast	70.7	Ref			Ref		
Other	76.8	1.09	0.96	1.23	1.10	0.97	1.24
Stage at diagnosis							
Local	79.4	Ref			Ref		
Regional	61.5	0.78	0.64	0.94	0.79	0.65	0.96
Distant	60.7	0.76	0.59	0.98	0.76	0.59	0.98
Not staged/unknown	82.5	1.04	0.82	1.32	1.02	0.80	1.30
Years since cancer diagnosis							
<2	77.1	Ref			Ref		
2‐<4	74.7	0.97	0.81	1.15	0.99	0.84	1.18
4+	73.5	0.95	0.80	1.13	0.97	0.82	1.15

^a^ Percent of women ages 50–74 reporting mammography within the past 2 years; percentages weighted to account for survey sample weighting and non‐response.

^b^ Model accounts for survey sample weighting and non‐response.

^c^ Multivariable model adjusted for Hispanic ethnicity, age at survey response, education, urban vs. rural residence, and years since diagnosis. Model accounts for survey sample weighting and non‐response.

Cervical cancer screening‐adherence varied significantly by age and educational attainment (Table 3). Women under age 45 at the time they were surveyed were more likely to be adherent to cervical cancer screening compared to women aged 55 and older (80.3% compared to 63.9%; aRR: 1.24; 95% CI: 1.03, 1.50). Survivors with some college reported greater adherence (76.4%) compared to individuals with high school or less education (54.0%; aRR: 1.43; 95% CI: 1.10, 1.87). Adherence was 69.4% among college graduates, but this was not significantly different from individuals with some college or those with high school or less education.

**TABLE 3:** Utah cancer survivors' adherence to cervical cancer‐screening guidelines by demographic, cancer diagnosis, and healthcare characteristics

	Percent adherent	Crude risk ratio (RR)^b^			Adjusted risk ratio (RR)^c^		
	%^a^	RR	95% Confidence interval		RR	95% Confidence interval	
			Lower	Upper		Lower	Upper
Hispanic ethnicity							
Hispanic	63.1	0.91	0.70	1.16	0.96	0.75	1.22
Non‐Hispanic	69.8	Ref			Ref		
Age							
Under 45	80.3	1.25	1.04	1.52	1.24	1.03	1.50
45–54	69.8	1.09	0.88	1.34	1.05	0.85	1.30
55+	63.9	Ref			Ref		
Education							
High school or less	54.0	Ref			Ref		
Some college or more	73.3	1.36	1.04	1.76	1.37	1.06	1.78
Geography							
Urban	67.7	Ref			Ref		
Rural	78.7	1.16	0.97	1.39	1.15	0.97	1.37
Area‐level proportion uninsured							
More uninsured	68.3	0.98	0.83	1.15	0.96	0.82	1.13
Fewer uninsured	70.0	Ref			Ref		
Health insurance							
Uninsured/unknown	42.0	0.59	0.30	1.17	0.55	0.27	1.12
Insured	70.8	Ref			Ref		
Financial hardships due to cancer							
Yes	66.2	0.90	0.77	1.06	0.84	0.71	1.00
No	73.5	Ref			Ref		
General health							
Good or better	70.8	Ref			Ref		
Fair or poor	60.4	0.85	0.64	1.13	0.90	0.68	1.21
Has primary care provider							
Yes	70.5	Ref			Ref		
No	61.7	0.88	0.65	1.17	0.84	0.63	1.11
Cancer site							
Cervical	54.9	Ref			Ref		
Other	69.8	1.27	0.63	2.58	1.16	0.55	2.45
Stage at diagnosis							
Local	69.3	Ref			Ref		
Regional	66.1	0.95	0.76	1.19	0.93	0.75	1.15
Distant	75.4	1.09	0.86	1.37	1.05	0.84	1.33
Not staged/unknown	81.2	1.17	0.77	1.79	1.09	0.73	1.63
Years since cancer diagnosis							
<2	62.9	Ref			Ref		
2‐<4	70.3	1.12	0.84	1.50	1.06	0.81	1.40
4+	70.2	1.12	0.84	1.49	1.12	0.86	1.47

^a^ Percent of women ages 21–65 reporting cervical cancer screening in accordance with guidelines; percentages weighted to account for survey sample weighting and non‐response.

^b^ Model accounts for survey sample weighting and non‐response.

^c^ Multivariable model adjusted for Hispanic ethnicity, age at survey response, education, urban vs. rural residence, and years since diagnosis. Model accounts for survey sample weighting and non‐response.

Adherence to colorectal cancer screening was significantly associated with survivor age, educational attainment, insurance status, and having a primary care provider (Table 4). Individuals under age 65 were less likely to participate in colorectal screening (63.1%) than those aged 65 or older (84.4%; aRR: 0.78; 95% CI: 0.66, 0.92). Survivors with some college (82.2%) or a college degree (83.7%) were more likely to be adherent to colorectal cancer‐screening guidelines compared to survivors with a high school education or less (72.1%). Compared to 81.7% of individuals with health insurance who reported adhering to colorectal cancer‐screening guidelines, those who were uninsured or had unknown insurance (25.4%) were less likely to have obtained recommended screening (aRR: 0.49; 95% CI: 0.27, 0.87). Survivors without a primary care provider had lower adherence to colorectal cancer‐screening guidelines than those with a primary care provider (45.6% vs. 83.0%, aRR: 0.57, 95% CI: 0.42, 0.79).

**TABLE 4:** Utah cancer survivors' adherence to colorectal cancer screening guidelines by demographic cancer diagnosis, and healthcare characteristics

	Percent adherent	Crude risk ratio (RR)^b^			Adjusted risk ratio (RR)^c^		
	%^a^	RR	95% Confidence interval		RR	95% Confidence interval	
			Lower	Upper		Lower	Upper
Sex							
Male	81.4	Ref			Ref		
Female	78.1	0.96	0.89	1.03	0.99	0.92	1.06
Hispanic ethnicity							
Hispanic	73.5	0.92	0.79	1.06	0.98	0.85	1.14
Non‐Hispanic	80.0	Ref			Ref		
Age							
50–54	63.1	0.75	0.63	0.89	0.78	0.66	0.92
55–64	77.7	0.92	0.85	1.00	0.92	0.86	1.00
65–74	84.4	Ref			Ref		
Education							
High school or less	72.1	Ref			Ref		
Some college or more	83.0	1.15	1.03	1.28	1.14	1.03	1.27
Geography							
Urban	80.0	Ref			Ref		
Rural	77.1	0.96	0.86	1.08	0.97	0.87	1.09
Area‐level proportion uninsured							
More uninsured	79.7	1.00	0.93	1.07	1.00	0.93	1.07
Fewer uninsured	79.6	Ref			Ref		
Health insurance							
Uninsured/unknown	25.4	0.31	0.16	0.60	0.49	0.27	0.87
Insured	81.7	Ref			Ref		
Financial hardships due to cancer							
Yes	78.7	0.98	0.91	1.06	0.99	0.92	1.07
No	80.3	Ref			Ref		
General health							
Good or better	79.3	Ref			Ref		
Fair or poor	81.6	1.03	0.93	1.14	1.06	0.96	1.16
Has primary care provider							
Yes	83.0	Ref			Ref		
No	45.6	0.55	0.40	0.75	0.57	0.42	0.79
Stage at diagnosis							
Local	80.2	Ref			Ref		
Regional	79.5	0.99	0.90	1.09	1.01	0.92	1.11
Distant	75.4	0.94	0.82	1.08	0.93	0.81	1.07
Not staged/unknown	83.3	1.04	0.85	1.27	1.00	0.81	1.23
Years since cancer diagnosis							
<2	79.1	Ref			Ref		
2‐<4	80.5	1.02	0.90	1.15	1.04	0.92	1.17
4+	79.0	1.00	0.88	1.13	0.99	0.88	1.12

^a^ Percent of survivors ages 50–75 reporting colorectal cancer screening in accordance with guidelines; percentages weighted to account for survey sample weighting and non‐response.

^b^ Model accounts for survey sample weighting and non‐response.

^c^ Multivariable model adjusted for Hispanic ethnicity, age at survey response, education, urban vs. rural residence, and years since diagnosis. Model accounts for survey sample weighting and non‐response.

## 4 DISCUSSION AND CONCLUSIONS

We evaluated adherence to cancer‐screening recommendations among cancer survivors using a population‐based sample of individuals diagnosed in Utah from 2012–2018. Results indicate that nearly 80% of Utah cancer survivors were up to date with recommended colorectal cancer screening in 2018–2020, which exceeds the Healthy People 2020 goal. Adherence to cervical cancer‐screening guidelines was just under 70%. Mammography screening adherence was 74%. While these rates are higher than some prior studies,^10^ our study finds that Utah cancer survivors did not meet Healthy People 2020 goals for cervical or breast cancer screening. Our analysis also found no significant changes in screening over the course of the three years of the study data. Due to their high risk of subsequent cancers, the fact that approximately 20–30% of survivors are non‐adherent for screening represents a critical care gap that warrants attention. There is opportunity to improve screening utilization in Utah for all three cancer types, and our findings demonstrate that efforts to improve adherence to screening recommendations in Utah should include the cancer survivor community.

Some prior research has found that cancer survivors are less likely than those without a history of cancer to adhere to certain cancer screenings.^27^, ^28^ Others have founds survivors receive more frequent screening.^29^ Our results indicate that Utah cancer survivors are more likely to obtain recommended colorectal cancer screening than the general population in Utah and the United States. In 2018, adherence to colorectal screening was 70.0% among the entire eligible Utah population and 69.5% nationwide.^30^ However, utilization of recommended breast and cervical cancer screening among Utah cancer survivors was similar to the Utah population at large and does not meet Healthy People 2020 goals. Prevalence of breast and cervical cancer screening in Utah falls below national averages. In 2018, 72.3% of all eligible Utah women reported adherence to breast cancer screening compared to 78.1% nationwide.^30^ Utah also ranks second‐to‐last in cervical cancer‐screening adherence among the US states at 72.5% compared to 79.9% nationwide.^30^

It is notable that colorectal cancer screening was more common than the other cancer screenings in the Utah cancer survivor population and that colorectal cancer screening among survivors in Utah is higher than that within the general population. A variety of plausible factors could explain these observations, including variations in the types of providers or health systems where survivors are receiving care relative to others, variation in knowledge and awareness of various cancers, or the fact that colonoscopy screening does not need to be repeated as frequently as mammograms or pap tests. Colorectal cancer screening has been steadily increasing over time in both Utah^20^ and the United States.^13^ The relatively higher rate of colorectal cancer screening among Utah cancer survivors may be reflective of this broader trend. Conversely, breast cancer screening adherence has held relatively stable and cervical cancer screening use has decreased over time in Utah and nationwide.^13^, ^20^ Evidence suggests that religiosity and beliefs regarding lack of susceptibility to cervical cancer among Utah women are associated with non‐adherence to HPV vaccination,^31^ and it is possible such beliefs could also affect adherence to cervical cancer‐screening guidelines.^32^

This study also found demographic disparities in adherence to screening guidelines among Utah cancer survivors. Similar to findings observed in a nationwide study of the U.S. population,^33^ we found that older age and lower educational attainment are associated with lower adherence to cervical screening. Age and education were also both associated with adherence to colorectal cancer screening, but in this instance younger individuals were less likely to be up to date with recommended screening. Among survivors of Hispanic ethnicity, the proportions screened for all three cancer types evaluated were similar to non‐Hispanic survivors. For breast cancer screening, we found that survivors in urban areas were less likely to be compliant with breast cancer‐screening recommendations than residents of rural areas. The percent of survivors adhering to cervical cancer‐screening guidelines was also lower in urban areas, but not significantly. We also found that some access to care variables were significantly associated with screening. Adherence to colorectal‐screening guidelines was lower for those with no insurance, Medicaid, or other insurance compared to Medicare. Also, survivors with a regular primary care provider were more likely to be in adherence with colorectal screening. Uninsured or unknown insurance type was also associated with a lower percentage of survivors obtaining screenings for all three types, but the difference was not significant for cervical and breast cancer.

Strengths of the present study were that it was population‐based, that it included survivors of all types of adult cancers, and that the survey achieved a strong response rate. This study's limitations include the reliance on self‐reported timing of most recent screening procedures, which could be subject to recall error. Additionally, because a large majority of Utah cancer survivors identify as non‐Hispanic white, we had too few participants of color to enable us to produce reliable estimates for other racial groups. Because cervical cancer‐screening guidelines apply only to women up to age 65, but a large proportion of cancer survivors are older than 65 years of age, the sample size for evaluating screening for cervical cancer in this study was relatively small, potentially affecting our ability to detect differences by demographic factors. Finally, we did not have all relevant health history information for participants, including factors which could have affected eligibility for screening such as prior receipt of a hysterectomy. We did not ask a question to ascertain if colorectal screening was performed after diagnosis, which would have allowed us to analyze colorectal cancer‐screening adherence among colorectal cancer survivors.

It is important that public health efforts to reduce disparities in cancer screening target the relevant barriers. The Affordable Care Act mandates coverage of these cancer screenings.^34^ Programs such as the Centers for Disease Control and Prevention's National Breast and Cervical Cancer Early Detection Program seeks to address financial and access barriers as well as increase public awareness in its efforts to improve screening among medically underserved populations.^35^ Efforts such as these may prove useful for the cancer survivors as well. This study demonstrates there is more work to be done to improve screening utilization among segments of the cancer survivor population. It may be necessary to develop cancer survivor‐specific interventions to improve screening outcomes. Our study identifies segments of the survivor population that are particularly less likely to adhere to screening recommendations. Further investigation into the barriers these survivors face, and interventions to address these barriers, are warranted. Using population‐based surveys, central cancer registries can collaborate with local cancer control programs to further identify and address the health needs of the growing population of cancer survivors.

## AUTHOR CONTRIBUTIONS

Morgan Millar: conceptualization, investigation, methodology, writing ‐ original draft, writing ‐ review and editing; Sandra Edwards: conceptualization, data curation, methodology, project administration, writing ‐ review and editing; Kim Herget: data curation, formal analysis, methodology, writing ‐ review and editing; Brian Orleans: data curation, formal analysis, methodology, writing ‐ review and editing; Blessing Ofori‐Atta: data curation, formal analysis, writing ‐ review and editing; Anne Kirchhoff: conceptualization, investigation, writing ‐ review and editing; Marjorie Carter: conceptualization, data curation, methodology, project administration, writing ‐ review and editing; Marie Nagata: conceptualization, writing ‐ review and editing; Carol Sweeney: conceptualization, funding acquisition, investigation, methodology, writing ‐ review and editing.

## FUNDING INFORMATION

This project was supported by the U.S. Centers for Disease Control and Prevention, Cooperative Agreement No. NU58DP006320. The Utah Cancer Registry is also supported by the U.S. National Cancer Institute‘s SEER Program, Contract No. HHSN261201800016I, with additional support from the University of Utah and Huntsman Cancer Foundation. This investigation was also supported by the University of Utah Study Design and Biostatistics Center, with funding in part from the National Center for Advancing Translational Sciences, National Institutes of Health, through Grant UL1TR002538.

## CONFLICT OF INTEREST

None.

## Supporting information


Table S1

Appendix S1

## Data Availability

The data that support the findings of this study are available on request from the corresponding author. Restrictions apply to the availability of these data, which are not publicly available due to privacy or ethical restrictions.
