# Supplementary material for: Adherence to Guideline‐Recommended cancer screening among Utah cancer survivors
Source: Cancer Med. 2022 Aug 27;12(3):3543–54. doi: 10.1002/cam4.5168 (PMC9939153; doi:10.1002/cam4.5168)
Supplement: Supplementary file 1 — Table S1 [file CAM4-12-3543-s002.docx]

Supporting File

Supplemental Table. Sample probabilities and sample frame

|  |  | Selected | % of eligible cases selected | Eligible number of cases selected from |
| --- | --- | --- | --- | --- |
| Year 1 | More uninsured | 533 | 4.0% | 13490 |
| Year 1 | less uninsured | 267 | 1.5% | 17569 |
| Year 2 | Areas with more uninsured residents, Hispanic | 111 | 9.7% | 1141 |
| Year 2 | Areas with more uninsured residents, Non-Hispanic | 439 | 3.5% | 12542 |
| Year 2 | Areas with fewer uninsured residents, Hispanic | 44 | 5.1% | 870 |
| Year 2 | Areas with fewer uninsured residents, Non-Hispanic | 306 | 1.8% | 16888 |
| Year 3 | Hispanic | 276 | 15.9% | 1731 |
| Year 3 | Non-Hispanic | 524 | 2.0% | 26048 |
