# Supplementary material for: Adherence to Guideline‐Recommended cancer screening among Utah cancer survivors
Source: Cancer Med. 2022 Aug 27;12(3):3543–54. doi: 10.1002/cam4.5168 (PMC9939153; doi:10.1002/cam4.5168)
Supplement: Supplementary file 2 — Appendix S1 [file CAM4-12-3543-s001.pdf]

Cancer Survivor Experiences Project
2020 Questionnaire

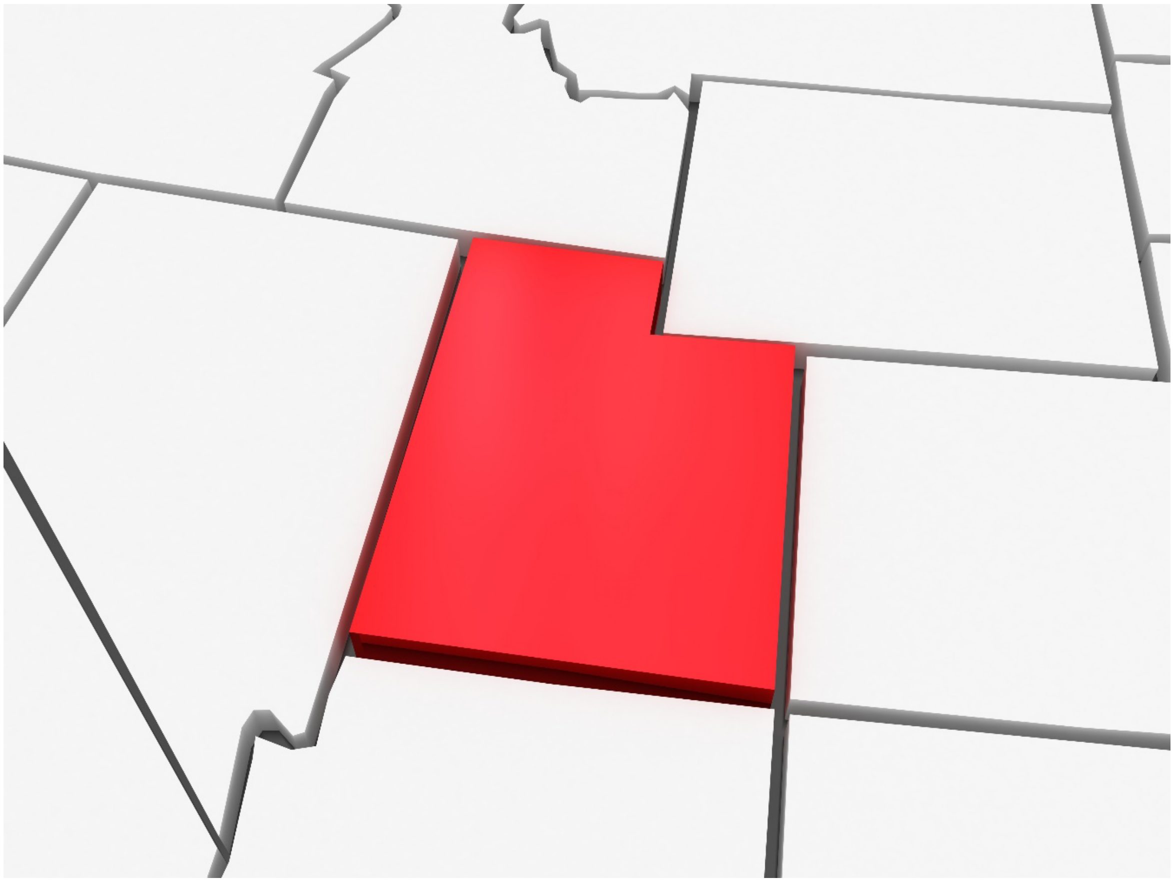

A project conducted by Utah Cancer Registry and Utah Department of Health

HEALTH
UNIVERSITY OF UTAH

Cancer Survivor Experiences Project Questionnaire

Start Here ▼

Thank you for sharing your experience as a cancer survivor by responding to this survey. The success of this project relies on the generous help of all of its participants. We very much value your insights.

When completing the questionnaire, please:

- Mark each box with an X or by completely filling in the box
- Completely erase or clearly mark any answers you wish to change
- Leave any question blank that you do not wish to answer
- Choose only one answer unless instructions indicate to 'select all that apply'
- Go directly to the next question unless you are instructed to skip to a certain question or to skip to the next section of questions

Please enter today's date.

☐☐ / ☐☐ / 2 0 ☐☐

m m *d d* *y y y y*

Section A Questions About Your General Health

These first questions ask about your health and life in general.

Q1. Would you say that in general your health is excellent, very good, good, fair, or poor? *Select one*

- ▢ Excellent
- ▢ Very good
- ▢ Good
- ▢ Fair
- ▢ Poor

Q2. Are you limited in any way in any activities because of physical, mental, or emotional problems? *Select one*

- ▢ Yes
- ▢ No

Q3. In general, how satisfied are you with your life? *Select one*

- ▢ Very satisfied
- ▢ Satisfied
- ▢ Dissatisfied
- ▢ Very dissatisfied

Section B Cancer Survivorship

The next few questions ask about your cancer treatment and follow-up care.

By cancer treatment, we mean:

- **Surgery including <u>removal</u> of uterus, thyroid, melanoma, cancerous polyps, lobe of a lung, or other tumor destruction or <u>removal</u> such as LEEP, laser, or cryosurgery**
- **Radiation therapy <u>not</u> including diagnostic CAT scans, MRIs, or diagnostic x-rays**
- **Chemotherapy or chemotherapy pills <u>not</u> including long-term hormonal therapy**

Q4. **Are you <u>currently</u> receiving treatment for cancer?** *Select one*

- ▢ Yes ➜*Skip to Q5, page 6*
- ▢ No, I haven't started treatment *Skip to Q5, page 6*
- ▢ No, I've completed active treatment (other than long-term hormonal therapy)
- ▢ No, I've declined treatment
- ▢ Treatment was not needed

Q4a. **Did any doctor, nurse, or other health professional <u>ever</u> give you a <u>written or printed summary</u> of all the cancer treatments that you received? By 'other healthcare professional', we mean a nurse practitioner, a physician's assistant, social worker, or some other licensed professional.** *Select one*

- ▢ Yes, received written or printed summary
- ▢ No, did NOT receive written or printed summary ➜*Skip to Q4c*
- ▢ Not sure ➜*Skip to Q4c*
- ▢ Does not apply to me ➜*Skip to Q4c*

Q4b. **Did you share, that is show or discuss, the <u>written or printed summary</u> of all the cancer treatments that you received with your primary care provider?** *Select one*

- ▢ Yes
- ▢ No
- ▢ Not sure

Q4c. **Have you <u>ever</u> received <u>written or printed instructions</u> from a doctor, nurse, or other health professional about where you should return or who you should see for routine cancer check-ups after completing your treatment for cancer?** *Select one*

- ▢ Yes, received written or printed instructions
- ▢ No, did NOT receive written or printed instructions ➜*Skip to Q5, page 6*
- ▢ Not sure ➜*Skip to Q5, page 6*

Q4d. **Did you share, that is show or discuss, the <u>written or printed instructions</u> about where you should return or who you should see for routine cancer check-ups after completing your treatment for cancer with your primary care provider?** *Select one*

- ▢ Yes
- ▢ No
- ▢ Not sure

Q5. **Did you participate in a clinical trial as part of your cancer treatment?** *Select one*

- ▢ Yes
- ▢ No
- ▢ Not sure
- ▢ Does not apply, I did not have cancer treatment

Q6. **Do you <u>currently</u> have physical pain caused by your cancer or cancer treatment?** *Select one*

- ▢ Yes
- ▢ No ➔*Skip to Q7*

Q6a. **Would you say your pain is currently under control…?** *Select one*

- ▢ With medication (or treatment)
- ▢ Without medication (or treatment)
- ▢ Not under control with medication (or treatment)
- ▢ Not under control without medication (or treatment)

Q6b. **Currently, which of the following do you <u>use to control your pain associated with cancer or cancer treatment</u>?** *Please read through the list carefully and select <u>all</u> that apply.*

- ▢ Opioids (Examples: codeine, fentanyl, hydrocodone, hydromorphone, morphine, oxycodone, tapentadol, tramadol)
- ▢ Nonopioid analgesics including nonsteriodal anti-inflammatory drugs and acetaminophen (Examples: aspirin, COX-2 inhibitors, ibuprofen, Advil, Motrin, Tylenol)
- ▢ Corticosteroids (Example: dexamethasone)
- ▢ Antidepressants and anticonvulsants
- ▢ Muscle relaxants (Examples: diazepam, lorazepam, metaxalone)
- ▢ Mind body therapies (Examples: biofeedback, breathing, hypnosis, mindfulness, relaxation)
- ▢ Psychosocial support
- ▢ Physical or occupational therapy
- ▢ Physical activity including walking, exercise, stretching, strength training, and yoga
- ▢ Local therapies including heat, cold packs, massage, medicated creams, ointments, and patches (topical lidocaine patch)
- ▢ Interventional procedures (Examples: electrical nerve stimulation, nerve blocks, neurotomy with radiofrequency ablation, and dorsal column stimulation)
- ▢ Acupuncture
- ▢ Medical cannabis (marijuana)
- ▢ I do not use anything to control my pain associated with cancer or cancer treatment
- ▢ Other form of pain management not listed above

Q7. **To the best of your knowledge, are you now free of cancer?** *Select one*

- ▢ Yes
- ▢ No
- ▢ Not sure

Section C Caregivers

This section is about caregivers, meaning friends or family members who may have provided help with getting to the doctor, going to appointments with you, making decisions about treatment, or providing other types of care and support during or after cancer treatment.

Please think about the time you were first diagnosed with cancer to now. If you have had more than one type of cancer, please think about your experiences across all of them.

Q8. **Since the time you were <u>first</u> diagnosed with cancer, has any friend or family member provided care to you during or after your cancer treatment?** *Select one*

- ▢ Yes
- ▢ No ➔*Skip to Section D*

Q8a. **Because of your cancer, its treatment, or the lasting effects of that treatment, did any of your caregivers <u>ever</u> take extended paid time off from work, unpaid time off, or make a change in their hours, duties or employment status?** *Select one*

- ▢ Yes
- ▢ No
- ▢ None of my caregivers were employed while caring for me
- ▢ Not sure

Section D Health Care Access

These next questions are about your access to health care and your ability to have that care paid for by insurance.

Q9. **Are you <u>currently</u> covered by any of the following types of health insurance or health coverage plans?** *Please select <u>all</u> that apply.*

- ▢ A plan purchased through an employer or union (includes plans purchased through another person's employer) ➔*Skip to Q10*
- ▢ A plan that you or another family member buys on your own ➔*Skip to Q10*
- ▢ Medicare ➔*Skip to Q10*
- ▢ Medicaid or other state program ➔*Skip to Q10*
- ▢ TRICARE (formerly CHAMPUS), VA, or Military ➔*Skip to Q10*
- ▢ Alaska Native, Indian Health Service, Tribal Health Services ➔*Skip to Q10*
- ▢ Some other source ➔*Skip to Q10*
- ▢ None (no coverage)

Q9a. **For how many months have you been uninsured?** *Select one*

- ▢ Less than 4 weeks
- ▢ Between 1 and 60 months (5 years) ➔*Please enter the number of months (1-60) here* ▢
- ▢ More than 5 years
- ▢ Not sure

Q10. **Were you <u>ever</u> denied health insurance or life insurance coverage because of your cancer?** *Select one*

- ▢ Yes
- ▢ No

Q11. **With your <u>most recent diagnosis of cancer</u>, did you have health insurance that paid for all or part of your cancer treatment? Note: health insurance also includes Medicare, Medicaid, or other types of state health programs.** *Select one*

- ▢ Yes
- ▢ No

Q12. **Do you have one person you think of as your personal doctor or health care provider?** *Select one*

- ▢ Yes, only one
- ▢ More than one
- ▢ No

Q13. **Was there a time in the past 12 months when you needed to see a doctor but could not because of cost?** *Select one*

- ▢ Yes
- ▢ No

Q14. **Was there a time in the past 12 months when you <u>did not take your medication as prescribed</u> because of cost? Do not include over-the-counter (OTC) medication.** *Select one*

- ▢ Yes
- ▢ No
- ▢ No medication was prescribed

Q15. **Since your cancer diagnosis, did you ever stay at a job in part because you were concerned about losing your health insurance?** *Select one*

- ▢ Yes
- ▢ No
- ▢ Does not apply to me

Q16. **Since your cancer diagnosis, did your spouse or significant other ever stay at a job in part because he or she was concerned about losing health insurance for the family?** *Select one*

- ▢ Yes
- ▢ No
- ▢ Does not apply to me

Q17. **About how long has it been since you last visited a doctor for a routine checkup? A routine checkup is a general physical exam, not an exam for a specific injury, illness, or condition.** *Select one*

- ▢ Within the past year (anytime less than 12 months ago)
- ▢ Within the past 2 years (1 year but less than 2 years ago)
- ▢ Within the past 5 years (2 years but less than 5 years ago)
- ▢ 5 or more years ago
- ▢ Not sure
- ▢ Never

Q18. In general, how satisfied are you with the health care you received? Would you say…? *Select one*

- ▢ Very satisfied
- ▢ Somewhat satisfied
- ▢ Not at all satisfied
- ▢ Does not apply to me

Q19. Please tell us some things that you believe are <u>missing from the care you are receiving as a cancer survivor</u>, that is to say your unmet needs.

Section E Medical Expenses

The next questions ask about different kinds of financial burden you or your family may have experienced because of your cancer, its treatment, or the lasting effects of the treatment.

Q20. <u>In the past 12 months</u>, have any of the following happened <u>because of medical expenses</u>?

Please mark "Yes" or "No" or "Not sure" for EACH financial burden.

	Yes	No	Not sure
A. Put off major purchases, such as a new home or car	▢	▢	▢
B. Been unable to pay for basic necessities like food, heat, or rent	▢	▢	▢
C. Had to take money out of savings	▢	▢	▢
D. Spent more than 10% of your income on medical expenses	▢	▢	▢
E. Had to borrow money	▢	▢	▢
F. Took on credit card debt	▢	▢	▢
G. Took out a mortgage against your home or took out a loan	▢	▢	▢
H. Thought about filing for bankruptcy	▢	▢	▢
I. Filed for bankruptcy	▢	▢	▢

Section F Cancer Screening

The next questions ask about health screenings you might have had.
Questions Q21, Q22, and Q22a are for <u>women</u>. <u>Men and women</u> should answers questions Q23-Q25.

Q21. *Women only* **A mammogram is an x-ray of each breast to look for breast cancer. When did you have your most recent mammogram?** *Select one*

- ▢ Within the past year (anytime less than 12 months ago)
- ▢ Within the past 2 years (1 year but less than 2 years ago)
- ▢ Within the past 3 years (2 years but less than 3 years ago)
- ▢ Within the past 5 years (3 years but less than 5 years ago)
- ▢ 5 or more years ago
- ▢ I have never had a mammogram
- ▢ Not sure

Q22. *Women only* **A Pap test is a test for cancer of the cervix. How long ago did you have your most recent Pap test?** *Select one*

- ▢ Within the past year (anytime less than 12 months ago)
- ▢ Within the past 2 years (1 year but less than 2 years ago)
- ▢ Within the past 3 years (2 years but less than 3 years ago)
- ▢ Within the past 5 years (3 years but less than 5 years ago)
- ▢ 5 or more years ago
- ▢ I have never had a Pap test ➔*Skip to Q23*
- ▢ Not sure ➔*Skip to Q23*

Q22a. *Women only* **An HPV test is sometimes given with the Pap test for cervical cancer screening. Did you have an HPV test with your most recent Pap test?** *Select one*

- ▢ Yes
- ▢ No
- ▢ Not sure

Q23. **A blood stool test is a test that may use a special kit at home to determine whether the stool contains blood. How long has it been since you had your last blood stool test using a home kit?** *Select one*

- ▢ Within the past year (anytime less than 12 months ago)
- ▢ Within the past 2 years (1 year but less than 2 years ago)
- ▢ Within the past 3 years (2 years but less than 3 years ago)
- ▢ Within the past 5 years (3 years but less than 5 years ago)
- ▢ 5 or more years ago
- ▢ I have never had a blood stool test
- ▢ Not sure

Q24. Sigmoidoscopy and colonoscopy are exams in which a tube is inserted in the rectum to view the colon for signs of cancer or other health problems. Have you ever had either of these exams? *Select one*

- ▢ Yes
- ▢ No ➔*Skip to Q25*
- ▢ Not sure ➔*Skip to Q25*

Q24a. For a sigmoidoscopy, a flexible tube is inserted into the rectum to look for problems. A colonoscopy is similar, but uses a longer tube, and you are usually given medication through a needle in your arm to make you sleepy and told to have someone else drive you home after the test. Was your most recent exam a sigmoidoscopy or a colonoscopy? *Select one*

- ▢ Sigmoidoscopy
- ▢ Colonoscopy
- ▢ Not sure

Q24b. How long has it been since you had your last sigmoidoscopy or colonoscopy? *Select one*

- ▢ Within the past year (anytime less than 12 months ago)
- ▢ Within the past 2 years (1 year but less than 2 years ago)
- ▢ Within the past 3 years (2 years but less than 3 years ago)
- ▢ Within the past 5 years (3 years but less than 5 years ago)
- ▢ Within the past 10 years (5 years but less than 10 years ago)
- ▢ 10 or more years
- ▢ Not sure

Q25. During the past 12 months, have you had either a flu shot or a flu vaccine that was sprayed in your nose? *Select one*

- ▢ Yes
- ▢ No

Section G Height and Weight

The next questions ask about your height and weight.

Q26. About how tall are you without shoes? *Please round fractions down.*

[ ] *feet* [ ][ ] *inches*

Q27. About how much do you weigh without shoes?

[ ][ ][ ]

Weight in pounds

Section H Health Behaviors

These questions ask about physical activity and smoking.

Q28. **<u>In a typical week</u>, how many days do you do any physical activity or exercise of at least moderate intensity, such as brisk walking, bicycling at a regular pace, or swimming at a regular pace?** *Select one*

- ▢ None → *Skip to Q29*
- ▢ 1 day per week
- ▢ 2 days per week
- ▢ 3 days per week
- ▢ 4 days per week
- ▢ 5 days per week
- ▢ 6 days per week
- ▢ 7 days per week

Q28a. **On the days that you do any physical activity or exercise of at least moderate intensity, how long do you typically do these activities?**

Please estimate your time in minutes <u>or</u> in hours. Write a number in <u>one</u> box below.

▢▢▢ *minutes per day*

▢ *hours per day*

Q29. **<u>In a typical week</u>, outside of your job or work around the house, how many days do you do leisure-time physical activities specifically designed to <u>strengthen your muscles such as lifting weights or circuit training</u> (do not include cardio exercise such as walking, biking, or swimming)?** *Select one*

- ▢ None
- ▢ 1 day per week
- ▢ 2 days per week
- ▢ 3 days per week
- ▢ 4 days per week
- ▢ 5 days per week
- ▢ 6 days per week
- ▢ 7 days per week

Q30. **Have you smoked at least 100 cigarettes in your entire life? Do not include: electronic cigarettes (e-cigarettes, njoy, bluetip), herbal cigarettes, cigars, cigarillos, little cigars, pipes, bidis, kreteks, water pipes (hookahs) or marijuana. Note: 5 packs = 100 cigarettes** *Select one*

- ▢ Yes
- ▢ No → *Skip to Section I on the next page*

Q30a. **Do you now smoke cigarettes every day, some days, or not at all?** *Select one*

- ▢ Every day
- ▢ Some days
- ▢ Not at all

Section I Social Connectedness

Next are some questions about your participation in community organizations. By community organization we mean any group that you might participate in such as church, sports leagues, self-help groups, service clubs, or professional organizations.

Q31. In the past 12 months, how often did you attend meetings of any organized group? *Select one*

- ▢ Never
- ▢ Less than once a year
- ▢ About once or twice a year
- ▢ Several times a year
- ▢ About once a month
- ▢ Every week
- ▢ Several times a week

Q32. In the past 12 months, about how often have you attended religious services? *Select one*

- ▢ Never
- ▢ Less than once a year
- ▢ About once or twice a year
- ▢ Several times a year
- ▢ About once a month
- ▢ Every week
- ▢ Several times a week

Q33. In the past 12 months, how often did you do volunteer work for religious, charitable, political, health-related, or other organizations? *Select one*

- ▢ Never
- ▢ Less than once a year
- ▢ About once or twice a year
- ▢ Several times a year
- ▢ About once a month
- ▢ Every week
- ▢ Several times a week

Q34. In the past 12 months, how often did you get together socially with friends or relatives? *Select one*

- ▢ Never
- ▢ Less than once a year
- ▢ About once or twice a year
- ▢ Several times a year
- ▢ About once a month
- ▢ Every week
- ▢ Several times a week

Section J Tell Us About You

Finally, we'd like to ask just a few more questions to make sure we are hearing from a variety of different people.

Q35. What is the <u>highest</u> grade or year of school you completed? *Select one*

- ▢ Never attended school or only attended kindergarten
- ▢ Grades 1 through 8 (Elementary)
- ▢ Grades 9 through 11 (Some high school)
- ▢ Grade 12 or GED (High school graduate)
- ▢ College 1 year to 3 years (Some college, Associate's Degree, or technical school)
- ▢ College 4 years or more (College graduate)

Q36. What is your <u>current</u> employment status? Include unpaid work in the family business or farm. *Please select <u>all</u> that apply*

- ▢ Working full-time (30 or more hours per week)
- ▢ Working part-time (less than 30 hours per week)
- ▢ Caring for home or family (not seeking paid work)
- ▢ Unemployed and looking for work
- ▢ Unable to work due to illness or disability
- ▢ Retired
- ▢ Student
- ▢ Other

Q37. Are you Hispanic, Latino/a, or Spanish origin? *Select one*

- ▢ Yes
- ▢ No

Q38. Which one or more of the following would you say is your race? *Please select <u>all</u> that apply*

- ▢ White
- ▢ Black or African American
- ▢ American Indian or Alaska Native
- ▢ Asian
- ▢ Native Hawaiian or Other Pacific Islander

If you have any other thoughts or comments that you would like us to know, please share them here.

Thank you for taking the time to answer these questions today. Please take a moment to review any questions you might have missed. On the next page, you'll find information about returning your questionnaire.
